# Hole-Selective SiN_*x*_ and AlO_*x*_ Tunnel Nanolayers for Improved Polysilicon Passivating Contacts

**DOI:** 10.1021/acsaem.4c01170

**Published:** 2024-11-14

**Authors:** Shona McNab, Audrey Morisset, Sofia Libraro, Ezgi Genç, Xinya Niu, Jack E. N. Swallow, Peter Wilshaw, Robert S. Weatherup, Matthew Wright, Franz-Josef Haug, Ruy S. Bonilla

**Affiliations:** † Department of Materials, University of Oxford, Oxford OX1 3PH, U.K.; ‡ School of Engineering, Ecole Polytechnique Fédérale de Lausanne, Neuchâtel CH-2000, Switzerland; § School of Photovoltaic and Renewable Energy Engineering, University of New South Wales, Sydney 2052, Australia

**Keywords:** hole-selective contacts, silicon solar cells, polysilicon contacts, silicon nitride, aluminum oxide, conduction mechanisms, passivation

## Abstract

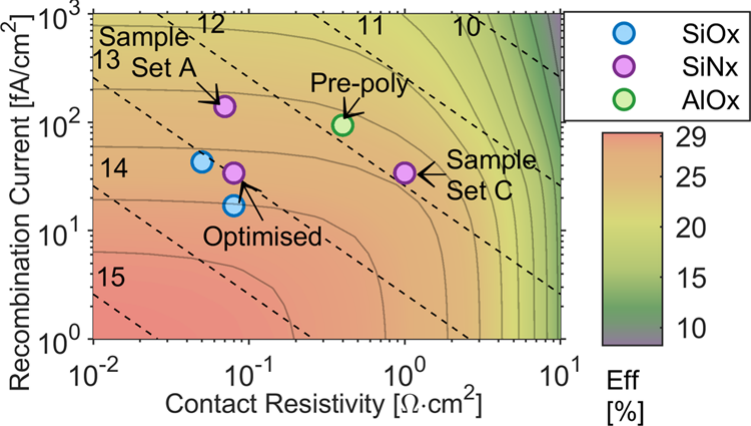

A highly efficient hole-selective passivating contact remains the crucial step required to increase the efficiency of polysilicon-based Si solar cells. The future development of solar modules depends on a device structure that can complement the electron-selective tunnel oxide passivating contact with an equivalent hole-selective contact. We investigate plasma enhanced chemical vapor deposited (PECVD) SiN_*x*_ and atomic layer deposited AlO_*x*_ as alternative nanolayers for the passivation layer in polysilicon tunnel contacts. We have fabricated p^+^ poly-Si contacts with resistivities below 100 mΩ·cm^2^ using these alternative metal oxide and nitride nanolayers. Initial passivation tests yielded low levels of passivation; however, a detailed understanding of the nanolayers elucidated the strategies to improve passivation significantly, achieving an implied open-circuit voltage (*iV*_OC_) of 698 mV and dark saturation current density (*J*_0_) of 34 fA/cm^2^ for a p^+^ poly-Si contact using a PECVD SiN_*x*_ interlayer. These are among the best reported for nitride-based nanolayer tunneling contacts, with research into nitride-based tunneling contacts being still in its infancy.

## 1 Introduction

Polysilicon-based contacts have emerged as a revolutionary contact technology unlocking dramatic increases in the efficiency of single-junction silicon solar cells using industrially compatible processes. The tunnel oxide passivating contact (TOPCon) structure, as it is known in the field, uses a SiO_*x*_ nanolayer topped with an n^+^ poly-Si as the electron contact with a diffused boron emitter for the hole contact.^1^ This structure has achieved high power conversion efficiencies, with efficiencies reaching 26%^2^ and it is now replacing the passivated emitter and rear cell (PERC) architecture in industrial manufacturing lines.^3^ Further increases in the efficiency of single-junction c-Si devices requires passivation of both the electron and hole contacts, with either both sides contacted^4−6^ or an interdigitated back contact (IBC) architecture.^4,6,7^ Studies of SiO_*x*_/poly-Si contacts consistently show higher dark saturation current density (*J*_0_) and contact resistivity (ρ_c_) for the hole contact compared to the electron contact.^6,8−12^ This can be attributed to the properties of the SiO_*x*_ and the Si/SiO_*x*_ interface. First, the valence band offset (VBO) of the SiO_*x*_ to Si is large, limiting the tunneling current.^13^ To obtain low-resistivity contacts, pinholes must be formed by high-temperature anneals. Pinholes are small areas of direct Si–Si interface, which are not passivated. At small pinhole densities, the passivation is not affected but if the pinhole density is above ∼10^6^ cm^–2^, the passivation quality of the contact is reduced.^14−16^ Second, in p-type poly-Si contacts the boron diffusion through the SiO_*x*_ layer can be excessively deep, increasing Auger recombination.^17^ In electron-selective poly-Si contacts, the phosphorus builds up at the poly-Si/SiO_*x*_ interface,^18^ resulting in a shallow diffusion and a strong field-effect passivation (FEP) induced by the sharp doping profile.^17^ On the other hand, boron has a high solubility in SiO_*x*_^19^ so it is not blocked at the SiO_*x*_ layer and a deep diffusion can form during the poly-Si anneal. This reduces the beneficial FEP that forms when a sharp dopant profile is present at the surface, as is the case for phosphorus-doped polysilicon. In addition, the boron within the SiO_*x*_ forms B–O pairs resulting in a strained Si/SiO_*x*_ interface^12,20^ and an increase in defect states. The high diffusivity of boron, combined with the requirement to form pinholes via a high annealing temperature, make the optimization of a hole TOPCon contact significantly more complex than the electron equivalent.

Recently, alternative dielectrics such as aluminum oxide (AlO_*x*_),^21−26^ silicon nitride (SiN_*x*_),^22,23,27^ and titanium oxide^22,28^ and hafnium oxide have been investigated for passivating contact structures. These materials offer potential benefits to SiO_*x*_ in hole-selective contacts. First, the dielectrics have a lower VBO, which enables low contact resistivity from purely tunneling conduction. The large VBO of 4.3 eV for SiO_*x*_,^23^ effectively blocks hole tunneling for thicknesses above 1.2 nm. SiN_*x*_ and AlO_*x*_ have lower VBO’s of 1.4 and 3.5 eV, respectively,^23^ corresponding to allowable tunneling thicknesses of 2.0 and 1.4 nm.^13,23^ Second, SiN_*x*_ or SiO_*x*_N_*y*_ interlayers have been shown to block boron effectively, and thus may produce improved diffusion profiles compared to SiO_*x*_ p^+^ poly-Si contacts.^17,29^ Finally, SiO_*x*_ has a low fixed charge density (*Q*_f_). It relies only on chemical passivation to provide a low surface recombination velocity. Alternative dielectrics can contain high *Q*_f_, which provides FEP in addition to chemical passivation. Simulations investigating the effect of interface charge show a beneficial effect in both the resistivity and passivation of the contacts for negatively charged tunnel layers. The charge can reduce the requirements for doping in the poly-Si as well. AlO_*x*_ is well-known to form a large negative charge at the Si surface.^23,30−33^ even in layers <10 ALD cycles (<1 nm).^23,31^ SiN_*x*_ is known to have a positive fixed charge,^33^ which could be detrimental to the properties of a p-type contact by reducing the passivation, or increasing the contact resistivity.^13^ Simulations have shown the highly doped p^+^ poly-Si layer compensates for positive charge, mitigating the effect for *Q*_f_ < 10^12^ q/cm^2^.^13^ Initial findings suggest this charge is sufficiently low in ∼2 nm films.^23^

Accurately quantifying the charge in the nanolayer dielectrics is difficult using standard techniques (e.g., ref (34,35)) due to the highly conductive nature of the films. Previous works^36,37^ used corona discharge to neutralize the internal charge in the dielectric. However, leakage current through the dielectric changes the net charge between deposition and measurement, resulting in an overestimation of *Q*_f_. Others used contacted *C*–*V* measurements but accounted for the leaky dielectric by incorporating the conduction through the dielectric, as well as the conductivity arising from interface states, into the theoretical models.^35,38,39^ Lastly, charge in the dielectrics can be characterized using surface photovoltage (SPV) measurements.^40^ In this technique, the samples are not contacted so leakage through the dielectric cannot occur. However, the effect of *Q*_f_ and the interface state density (*D*_it_) on the measured signal cannot be completely isolated, which limits precision. Developing techniques to determine the fixed charge in nanolayer dielectrics is crucial to maximize the potential of these materials in passivating contact structures.

There have been few studies incorporating dielectrics other than SiO_*x*_ into poly-Si contacts. The wealth of studies on SiO_*x*_ has enabled optimized processing conditions to be established for SiO_*x*_ and hole-selective contacts have been fabricated with *J*_0_ approaching 5 fA/cm^2^ for p^+^ poly-Si on p-type wafers.,^41^ while p^+^ poly-Si on n-type wafers can generally obtain better passivation,^11,20,42^ with the lowest reported *J*_0_ being 1.5 fA/cm^–2^.^43^ For non-SiO_*x*_ dielectrics with p^+^ poly-Si on p-Si, the best SiN_*x*_ contacts have measured an *iV*_oc_ of 690 mV^22^ (approximate *J*_0_ of 73 fA/cm^2^ extracted using ref (44)) with a ρ_c_ of 800 mΩ·cm^2^,^22^ and AlO_*x*_ contacts have reported a *J*_0_ of 14 fA/cm^2^ with a ρ_c_ of 300 mΩ·cm^2^.^37^ An alternative approach is to dope a SiO_*x*_ layer with nitrogen using an N_2_O plasma.^17,45^ This method aims to combine the beneficial boron blocking seen in nitride-based contacts with the chemical passivation of an SiO_*x*_ nanolayer. It has proved effective, with a low *J*_0_ of 6 fA/cm^2^ measured for p^+^poly-Si on n-Si.^46^

For a full area passivating contact to have a negligible impact on the series resistance of the cell, the contact resistivity must be below 100 mΩ·cm^2^.^47^ So far, it has proved challenging for SiN_*x*_ and AlO_*x*_ contacts to meet this benchmark^22,37^ while maintaining high levels of passivation, despite the potential theoretical advantages over SiO_*x*_. However, many aspects of these nanolayers are yet to be fully understood, so there is still scope for significant improvement with optimized processing.

In this study, SiN_*x*_ and AlO_*x*_ are investigated in hole-selective poly-Si contacts. The resistivity and passivation quality of the nanolayers are compared to UV–O_3_ SiO_*x*_ reference samples. In addition to measuring the contact properties of the alternative nanolayers, there is a focus on obtaining a detailed understanding of the mechanisms governing the passivation and transport properties of the contacts. This is achieved through temperature-dependent current–voltage measurements (T–J–V) to establish the dominant conduction mechanisms, and by developing a capped *C*–*V* analysis to determine the relative contribution from chemical and field-effect passivation. Using the understanding gained from these techniques, we processed a new set of samples with improved passivation. They showed a large scope for process optimization and efficiency improvements when the tunneling dielectric material is reconsidered in p-TOPCon.

## 2 Experimental Methodology

### 2.1 Sample Fabrication

**Figure 1:**
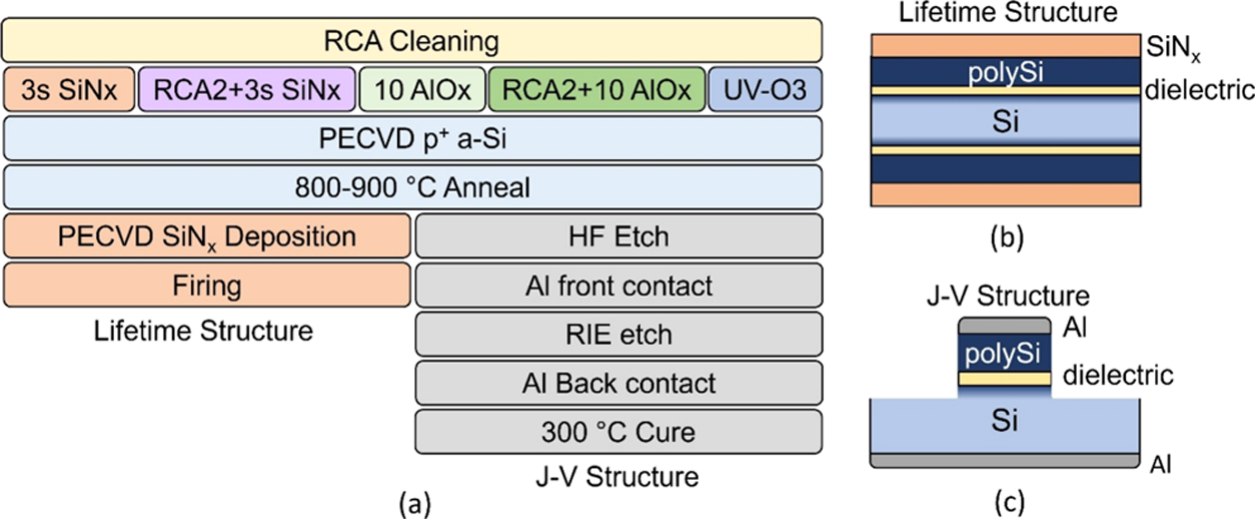
(a) Sample preparation process flow. A schematic of the final sample structure for (b) lifetime and PL measurements and (c) *J*–*V* measurements.

Poly-Si passivating contacts are prepared using the process flow shown in Figure 1a. Float-zone p-type wafers (2 Ω·cm) are cleaned using standard RCA cleaning and dipped in hydrofluoric acid (HF) before dielectric nanolayer deposition of RF PECVD (RF PECVD) silicon nitride or atomic layer deposition (ALD) aluminum oxide. In an additional group, the samples undergo a second treatment in RCA2 solution after HF etching, and the resulting silicon oxide layer forms an interlayer between the silicon and deposited dielectric (SiN_*x*_ or AlO_*x*_). Silicon nitride is deposited using an Oxford Instruments PECVD at 350 °C. The chamber pressure is held at 650 mTorr while 20 sccm silane, 40 sccm ammonia, and 980 sccm N_2_ are admitted into the chamber. A 20 W plasma is initiated for 3 s to deposit an SiN_*x*_ layer of ∼2 nm. Aluminum oxide is deposited in an Anric thermal ALD at 150 °C. One full AlO_*x*_ cycle consists of 3× trimethylaluminum pulse/purge cycles followed by 2× H_2_O pulse/purge cycles. Ten deposition cycles (10C) form a ∼1.5 nm AlO_*x*_ layer. The reported thicknesses were measured using ellipsometry with a FilmSense FS1 multiwavelength ellipsometer using a Cauchy fitting for SiN_*x*_ and the model of McGraw-Hill for AlO_*x*_.^48^

For fabrication of p+ poly-Si contacts, a boron-doped amorphous silicon (a-Si) layer is deposited on top of the thin passivating dielectric by PECVD in a KAI-M system from Unaxis. The layer stack is then annealed in an Ar atmosphere with a heating rate of 10 °C/min to the set point, immediately followed by a 2 °C/min cooling. Such annealing results in the crystallization of the a-Si layer into poly-Si, the activation of B dopants in the layer, and shallow B-diffusion in the Si substrate. Optimization of the PECVD parameters for a-Si deposition and annealing temperature was carried out in previous work to maximize the surface passivation while minimizing the contact resistivity of the resulting p+ poly-Si contact.^49^ The poly-Si contact is formed on both sides of the Si substrate for lifetime samples. Then, a double-sided PECVD SiN_*x*_ layer of about 80 nm is deposited and fired at 840 °C to provide hydrogenation (Figure 1b). The p^+^ poly-Si has previously been shown to achieve a high *iV*_OC_ in SiO_*x*_ poly-Si structures.^49,50^ The SiN_*x*_ is removed using an 5% HF solution for electrochemical capacitance–voltage (ECV) and resistivity measurements. Due to slight variations in the processing conditions of the dielectrics and poly-Si layers, a SiO_*x*_ reference sample is included in each processing batch. The SiO*_x_* layer is grown on an HF dipped wafer by exposure to UV light for 2 min (Jelight UVO cleaner 42) to form a 1 ± 0.3 nm layer. Table 1 details the dielectric layers and poly-Si anneal temperature included in each sample set.

**Table 1:** Dielectric Interlayers and Poly-Si Anneal Temperatures for Each Sample Set

	Dielectric Interlayers	Poly-Si Anneal Temperature (°C)
Sample Set A	3 s SiN_*x*_	850
	10 c AlO_*x*_	
	RCA2 + 3 s SiN_*x*_	
	RCA2 + 10 c AlO_*x*_	
	UV–O_3_	
Sample Set B	3 s SiN_*x*_	800, 850, 900
	UV–O_3_	
Sample Set C	RCA2 + 3 s SiN_*x*_	800, 850, 900
	RCA2 + 10 c AlO_*x*_	
	UV–O_3_	

The resistivity of the dielectric nanolayers is measured by depositing Al contacts using a thermal evaporator. The poly-Si is etched using reactive ion etching (RIE) to leave an exposed contact stack, preventing current spreading through the highly doped poly-Si. The rear poly-Si is also removed using RIE, and then a rear Al contact is deposited. The contacts are cured on a hot plate at 300 °C for 10 min. The final structure for resistivity measurements is shown in Figure 1c.

### 2.2 Characterization

The minority carrier lifetime was measured on symmetrical samples with either a Sinton WCT-100 or WCT-120 instrument. The implied open-circuit voltage (*iV*_OC_) was extracted at 1 sun conditions and the *J*_0_ for a single side is extracted using the method described in ref (51). Photoluminescence (PL) images are taken with an in-house built setup. The photocarriers are generated using an 808 nm wavelength laser and a PIXIS camera from Princeton Instruments is used for the imaging. The ECV measurements are performed using an ECV CVP21 tool from WEP. A 0.1 mol/L NH_4_F solution is used as the etchant. X-ray photoelectron spectroscopy (XPS) is used for compositional analysis and band alignments of the nanolayer dielectrics to silicon. Full details of the XPS are included in the Supporting Information.

Current density–voltage (*J*–*V*) measurements were performed using a Keithley 2611 source meter, with the sample structure shown in Figure 1c. The RIE etch isolates the contact stack to prevent lateral conduction in the poly-Si layer. A straight *J*–*V* curve between ±0.1 V indicated ohmic characteristics for all samples. The total resistivity (ρ_tot_) is extracted from the inverse gradient of the curve between ±0.01 V as
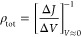
and the contact resistivity (ρ_c_) is determined by removing the spreading resistance of the wafer (*R*_s_), given by,
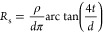
Where ρ is the wafer base resistivity, *d* is the contact diameter and *t* is the wafer thickness. Hence, the contact resistivity is calculated as
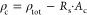
Where *A*_c_ is the contact area. Temperature-dependent *J*–*V* measurements are used to determine the dominant hole transport mechanisms across the dielectric interlayers. The measurements followed the procedure detailed in previous work.^23^

#### 2.2.1 Capped Capacitance–Voltage (*C*–*V*)

**Figure 2:**
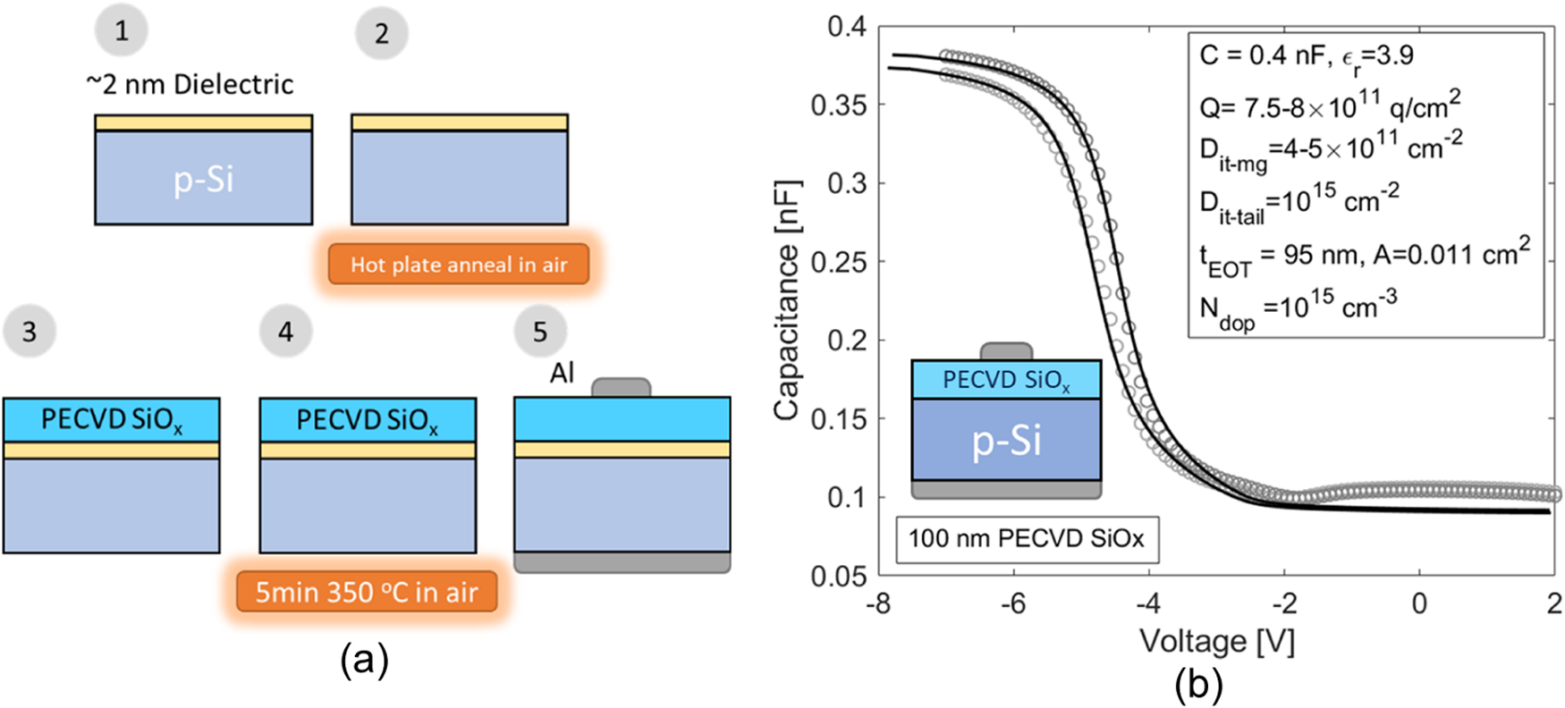
(a) Sample preparation for *C*–*V* analysis. (1) Deposition of dielectric, (2) hot plate anneal (if required), (3) deposition of PECVD SiO*_x_*, (4) 350 °C anneal of PECVD SiO_*x*_ (5) deposited Al contacts on front and rear. (b) *C*–*V* curve of 100 nm PECVD SiO_*x*_ with no dielectric interlayer (two contacts measured). Insets show sample schematic and theoretical fitting parameters.

To determine the interface properties of the highly conductive tunnel dielectrics, a capped *C*–*V* method was developed using a low-damage PECVD 100 nm SiO_*x*_ layer to prevent conduction through the dielectrics. The SiO_*x*_ is deposited using Oxford Instruments PlasmaPro 80. The chamber is held at 800 mTorr with gas flows of 10 sccm silane, 680 sccm N_2_O, and 790 sccm N_2_. The stage is set to 350 °C and a plasma power of 20 W is used to deposit the SiO_*x*_ at a rate of ∼1 nm/s. The thick SiO_*x*_ is deposited on top of the nanolayer dielectrics to suppress the high conductivity, while the original Si/dielectric interface strongly influences the *C*–*V* signal. Silicon oxide prevents a substantial FEP effect, which would affect the measurements. Some hydrogenation may occur during the SiO_*x*_ layer deposition and reduce the *D*_it_. Indeed, the lifetime of the SiN_*x*_ samples increased after SiO_*x*_ deposition (from ∼40 to ∼100 μs). Figure 2a shows the process flow for fabricating the capped *C*–*V* test samples.

*C*–*V* measurements are performed on 10 Ω·cm, p-type, Cz wafers at 1 MHz using 1 mm circular aluminum contacts. An Agilent E4980A precision LCR meter is used to sweep the voltage from the depletion region to accumulation with a 20 s initialization delay and a 1 s step delay. The A.C. signal is set to 0.05 V. Theoretical fitting of the *C*–*V* curve is used to extract the *Q*_f_ and *D*_it_ of the Si/dielectric interface.^52^ The density of interface states as a function of the band gap energy is modeled as a constant value at the mid gap and an exponentially increasing density toward the band edges.^53^ The density of states at the mid gap is used when reporting values of *D*_it_.

Figure 2b shows a *C*–*V* curve for a 100 nm PECVD SiO_*x*_ sample, without a nanolayer dielectric interlayer, and includes the theoretical fit (black line). The sample shows a characteristic “S shaped” curve, as expected for a metal–insulator–semiconductor (MIS) device. The voltage at which the inflection point occurs indicates the charge present at the Si/dielectric interface, while the transition between the accumulation and depletion regimes provides information on the density of interface states. The charge and *D*_it_ indicated in the inset are higher than would be expected for a 100 nm thermal SiO_*x*_. The high *D*_it_ is as unsurprising for a low-temperature deposition of SiO_*x*_. When measuring the nanolayer dielectrics, the PECVD SiO_*x*_ does not directly contact the silicon, so the *C*–*V* measurement is not affected by the SiO_*x*_*D*_it_. The charge of (7–8) × 10^11^ q/cm^2^ might have a minor influence on the underlying dielectric, resulting in a small systematic error in the absolute value of *Q*_f_ extracted. The charge likely forms at the Si/PECVD SiO_*x*_ interface, which is not present when PECVD SiO_*x*_ is used as a capping layer on another dielectric. Therefore, it cannot be corrected by simply subtracting the charge in the PECVD SiO_*x*_ from the value measured in the dielectric/SiO_*x*_ stack. The influence of this charge is considered in the discussion of the results. The error is systematic, so it is consistent for all measurements, and therefore, the comparison between samples is unaffected.

## 3 Results and Discussion

### 3.1 Resistivity Measurements

**Figure 3:**
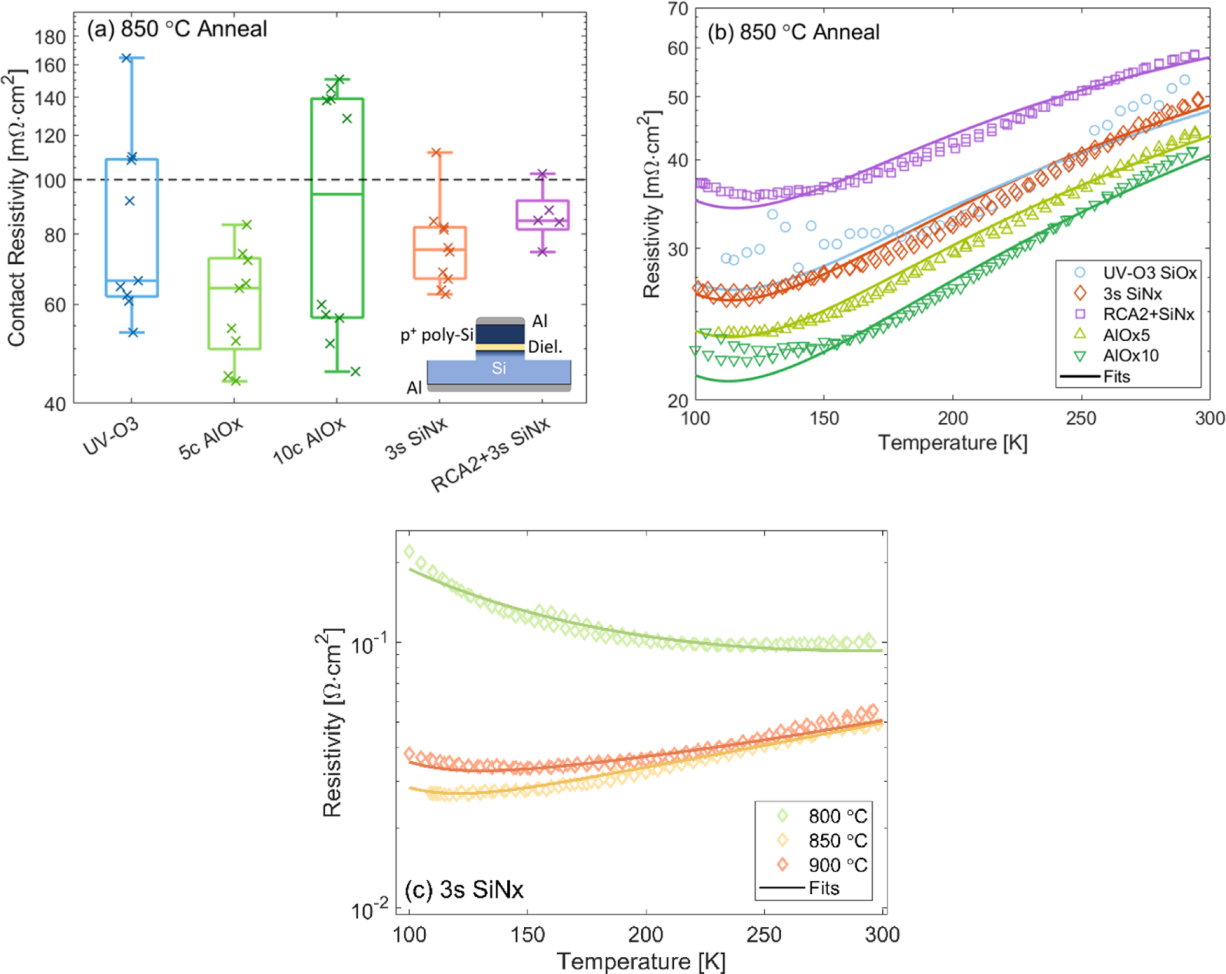
(a) Contact resistivity of Sample Set A: UV–O_3_ SiO_*x*_, 5 and 10 cycles of ALD AlO_*x*_, 3 s PECVD SiN_*x*_, and RCA2 + 3 s PECVD SiN_*x*_ nanolayers annealed at 850 °C. The box plots indicate the median, 25th and 75th percentiles, and the maximum and minimum values. A schematic of the samples is shown in the inset. (b) The total resistivity measured between 100 and 300 K with theoretical fits for Sample Set A. (c) The total resistivity measured between 100 and 300 K with theoretical fits for Sample Set B for different annealing conditions. A pinhole radius of 0.5 nm is assumed.

Poly-Si contacts with either SiN_*x*_ or AlO_*x*_ nanolayers were fabricated according to the process in Figure 1a (Sample Set A) and the resistivity was measured as described in Section 2.2. The contact resistivity was measured for UV–O_3_ SiO_*x*_, 5 and 10 cycles of ALD AlO_*x*_, 3 s PECVD SiN_*x*_, and RCA2 + 3 s PECVD SiN_*x*_ nanolayers. The poly-Si was annealed at 850 °C for all samples, and the measured contact resistivity is shown in Figure 3a. The samples with 5 cycles of AlO_*x*_ or 3 s SiN_*x*_ have comparable resistivity to the UV–O3 SiO_*x*_. A slight increase in the resistivity is measured for the 10 cycles of AlO_*x*_ and the RCA2 + SiN_*x*_ stacks but all contacts stacks have median resistivities below 100 mΩ·cm^2^. A contact resistance of 100 mΩ·cm^2^ is a useful benchmark for fabricating a solar cell with a full area contact, without a significant reduction in the fill factor (∼0.05%_abs_).^47^

Temperature-dependent *J*–*V* measurements were also performed for the dielectrics to provide information on the conduction mechanisms present in the samples. Figure 3b shows the total resistivity, ρ_tot_, measured between 100 and 300 K. The solid lines are theoretical fits, with the fitting parameters shown in Table 2. The equivalent circuit diagram and an example fitting are shown in Supporting Information Section 1, while a detailed description of the fitting procedure and the experimental determination of the VBO using the XPS based Kraut’s method, is described in previous work.^23,54^ The instrumental set up is described in Supporting Section 3. It can be seen in Figure 3b that all dielectrics follow the same trend with temperature. The decrease in resistivity with reducing temperature is indicative of pinhole dominated conduction, and indeed high pinhole concentrations are required to fit the data, as shown in Table 2. A second batch of samples was prepared to study the effect of the annealing temperature on the transport mechanisms (Sample Set B). The 3 s SiN_*x*_/poly-Si samples were annealed at 800, 850, and 900 °C. For the 3 s SiN_*x*_ layer after an 800 °C anneal, the T–J–V curve shows the opposite behavior with an increase in the resistivity at low temperature. This is fitted with purely tunneling conduction, indicating the pinhole density is below the sensitivity of the technique and that these layers allow substantial tunneling conduction despite being about 1.9 nm thick. It is noted that the 800 °C sample measures a slightly thicker dielectric. If this discrepancy occurred during SiN_*x*_ deposition, the thicker dielectric may have been less susceptible to forming pinholes during anneal, which, in combination with the lower temperature, resulted in the tunneling behavior and observed temperature trend. Alternatively, the higher temperature anneals could induce thinning of the SiNx (densification) as well as forming pinholes. We cannot distinguish between these two possibilities, but the primary conclusion still stands. It is possible to fabricate purely tunneling hole-selective poly-Si contacts. This means the high-temperature anneal may to be optimized solely for passivation. In the contacts that formed pinholes, there is a mixed mode of current transport, with contributions from tunneling and pinholes. This is highlighted in Figure S1, where it is shown that our samples are dominated by tunneling conduction at room temperature, while at low-temperature pinhole conduction dominates. Hence, although pinholes form during the high-temperature processing, these pinholes are not required to achieve the low-resistivity SiN_*x*_ contact. The high pinhole concentration results in a larger area of direct Si–Si contact, which can reduce the contact passivation quality. An increase in *J*_0_ is predicted at pinhole densities > 10^6^ cm^–2^^14−16,55^—a threshold that is significantly exceeded in these samples.

**Table 2:** Fitting Parameters for T–J–V Measurements of Sample Set A and B, Assuming a Pinhole Radius of 0.5 nm

	Dielectric	*T* [°C]	VBO [eV]	*t*_diel_ [nm]	Φ_b_ [eV]	*N*_pin_ [×10^8^ cm^–2^]
Set A	UV–O_3_ SiO_*x*_	850	4.3	1.2	0	1
	3 s SiN_*x*_	850	1.4	1.9	0	1.1
	RCA2 + 3 s SiN_*x*_	850	1.4	1.92	0	0.6
	5 cycles AlO_*x*_	850	3.5	1.32	0	1.4
	10 cycles AlO_*x*_	850	3.5	1.32	0	2
Set B	3 s SiN_*x*_	800	1.4	1.86	0	0
	3 s SiN_*x*_	850	1.4	1.73	0	1
	3 s SiN_*x*_	900	1.4	1.73	0	0.6

### 3.2 Passivation

**Figure 4:**
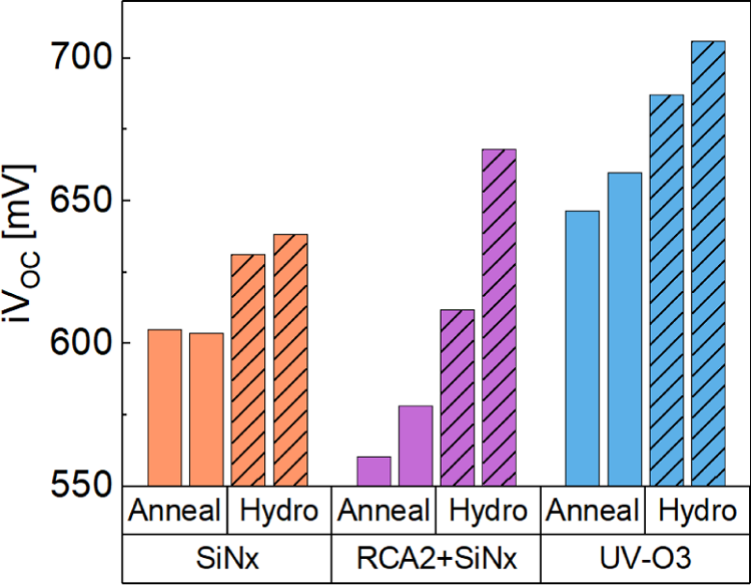
*iV*_OC_ of Sample Set A, SiN_*x*_ nanolayers compared to the UV–O_3_ SiO_*x*_ control samples. Samples were annealed at 850 °C (shown as Anneal) then hydrogenated (shown as Hydro) using a fired PECVD SiN_*x*_. Two samples are measured at each processing step.

The passivation quality of the poly-Si contacts was initially investigated using QSSPC on symmetrical lifetime samples of Sample Set A. The samples were measured after the 850 °C anneal and again after a PECVD SiN_*x*_ hydrogenation layer was deposited on top of the poly-Si and fired at 840 °C. At this stage, the AlO_*x*_ samples had a lifetime too low to be measured. The SiN_*x*_ samples are compared to the UV–O_3_ SiO_*x*_ reference via their *iV*_OC_, as pictured in Figure 4. The passivation of the SiN_*x*_ layers is currently lower than the SiO_*x*_ reference, both after annealing and hydrogenation. The RCA2 + SiN_*x*_ shows the most significant improvement in *iV*_OC_ after hydrogenation, with one sample reaching an *iV*_OC_ of 668 mV. We note that for this batch, the *iV*_OC_ measured on samples featuring the UV–O_3_ SiO_*x*_ reference was about 15–20 mV lower than usual after annealing and hydrogenation, with optimized UV–O_3_ SiO_*x*_ poly-Si layers previously reaching *iV*_OC_ of 720 mV.^56^ We attributed the variation to a drift in the PECVD chamber conditioning when depositing the poly-Si layer. This suggests that the *iV*_OC_ measured on samples featuring thin SiN_*x*_ and AlO_*x*_ layers may also be higher when combined with optimal poly-Si processing.

**Figure 5:**
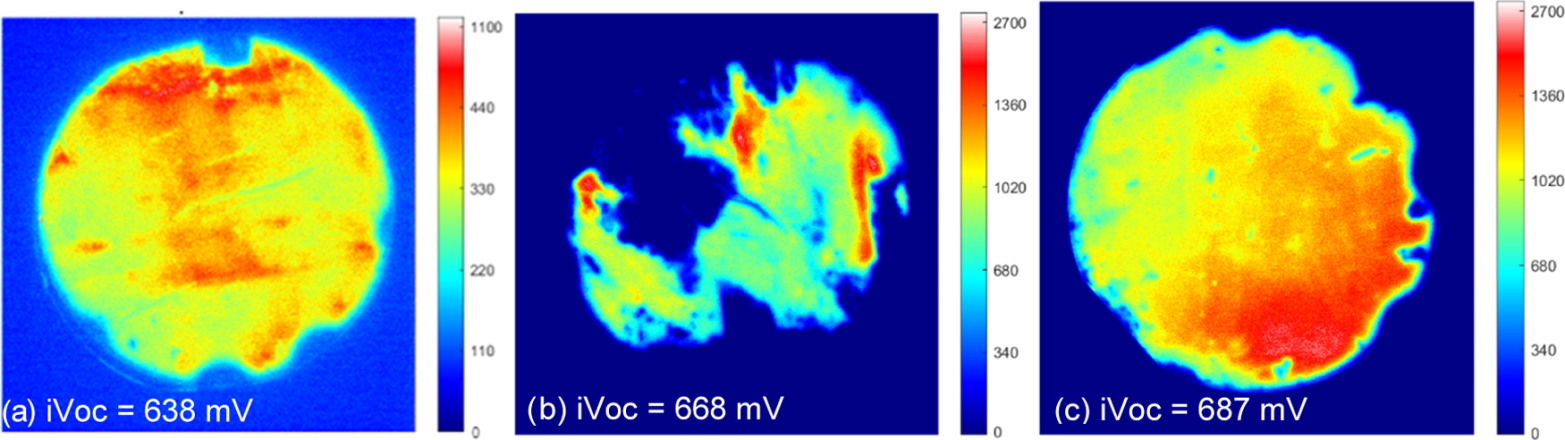
Photoluminescence images of (a) SiN_*x*_, (b) RCA2 + SiN_*x*_, and (c) UV–O_3_ SiO_*x*_ after hydrogenation.

PL images of the poly-Si samples after hydrogenation provide a spatial map of the passivation quality. Figure 5 shows PL images of (a) SiN_*x*_, (b) RCA2 + SiN_*x*_, and (c) UV–O_3_ SiO_*x*_ samples after the hydrogenation treatment. Images of the other samples are shown in Figure S3. The UV–O_3_ SiO_*x*_ has uniform passivation, while significant inhomogeneities are observed in both SiN_*x*_ samples. The SiN_*x*_ samples have large areas with poor passivation, which could be due to issues with sample handling or a nonuniform deposition of SiN_*x*_. In the RCA2 + SiN_*x*_ sample, the regions with the best passivation quality approach that of the SiO_*x*_. The significant spatial variation partially explains the low *iV*_OC_ observed in the SiN_*x*_ samples, as the measured *iV*_OC_ is an average across the area of the Sinton instrument sensor. Although, the brighter regions in the SiN_*x*_ do not quite represent the same *iV*_OC_ as the control UV/O_3_ sample.

#### 3.2.1 *D*_it_ and *Q*_f_ Analysis Using Capped *C*–*V*

**Figure 6:**
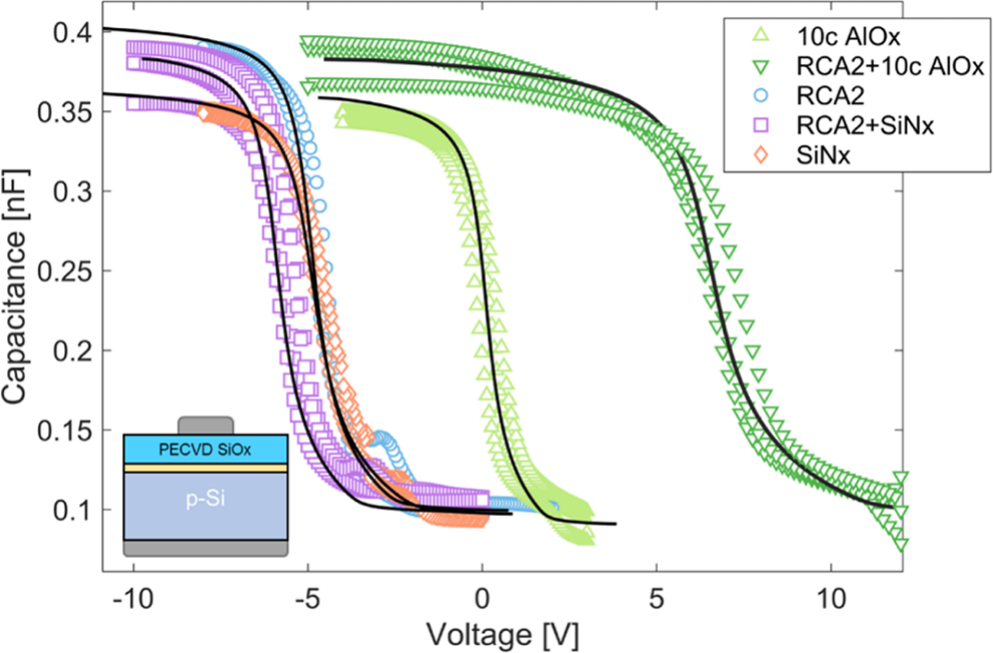
*C*–*V* analysis of AlO_*x*_ and SiN_*x*_ nanolayer stacks. Solid lines indicate the theoretical fitting, with fitting parameters detailed in Table 3. A schematic of the sample structure is shown in the inset.

The reasons for the poor passivation quality of the AlO_*x*_ and SiN_*x*_ were investigated further to develop a greater understanding of the differences between these dielectrics and a standard SiO_*x*_ layer. One of the key differences is that SiN_*x*_ and AlO_*x*_ can have high interface charge densities. This is well documented in thick AlO_*x*_ and SiN_*x*_ layers used for surface passivation.^33^ Capacitance–voltage measurements are often used to extract the interface charge and density of defect states at a semiconductor/dielectric interface.^57,58^ However, the high conductivity of the nanolayers used in passivating contacts prevents an accurate capacitance from being measured. To overcome this problem a novel technique was developed, where a thick PECVD SiO_*x*_ (∼100 nm) was deposited on top of the nanolayer dielectric. This reduces the conductivity while the measurement remains highly sensitive to the Si/nanolayer interface. Figure 6 shows the *C*–*V* curves for nanolayer dielectrics capped with 100 nm PECVD SiO_*x*_. SiN_*x*_ and AlO_*x*_ layers are compared to an RCA2 silicon oxide. All samples were made before any polysilicon was deposited or fired, and thus are only indicative of the initial condition the interface nanolayers display. The black lines indicate theoretical fits, and the fitting parameters are detailed in Table 3.

**Table 3:** Fitting Parameters for Capped *C*–*V* Measurements

Dielectric	*Q*_f_ [10^12^ q/cm^2^]	*D*_it_ [10^12^ cm^–2^]
RCA2 SiO_*x*_	0.7 ± 0.1	0.5 ± 0.2
3 s SiN_*x*_	0.6 ± 0.05	0.6 ± 0.2
RCA2 + 3 s SiN_*x*_	1 ± 0.1	0.4 ± 0.2
10 c AlO_*x*_	–0.4 ± 0.1	0.4 ± 0.2
RCA2 + 10 c AlO_*x*_	–3.2 ± 0.2	2 ± 0.5

In Figure 6, the SiN_*x*_ nanolayer shows a similar behavior to the RCA2 SiO_*x*_, and Table 3 confirms similar values of both *Q*_f_ and *D*_it_. A slight increase in positive charge is seen for the RCA2 + SiN_*x*_ layer. Note that there is a small systematic shift to positive *Q*_f_ due to the intrinsic charge in PECVD SiO_*x*_ (as discussed in Section 2.2.1 and shown in Figure 2b). Despite this, *Q*_f_ below 10^12^ cm^–2^ was observed in all SiN_*x*_ layers, and previous simulation work determined that at this charge concentration, there is only a small detrimental effect in p^+^ poly-Si contact properties.^13^ AlO_*x*_ shows a distinct shift to negative charge, increasing significantly with the addition of an RCA2 interlayer. At such high *Q*_f_, the negative charge can contribute to reductions in ρ_c_ and *J*_0_ of the contact.^13^ The higher *Q*_f_ observed for the RCA2 + AlO_*x*_ stack relates to the reactivity of the ALD precursors to the silicon surface. The oxidized surface improves the adsorption of the TMA,^59^ resulting in a higher concentration of Al in the film. This is observed via stoichiometry analysis using XPS (Figure S7). The *D*_it_ increase in the RCA2 + AlO_*x*_ stack is unexpected and could inhibit higher passivation levels. Further investigation is required to study the cause of the high *D*_it_ and develop methods to mitigate it. These results agree with previous measurements using SPV,^23^ further validating the technique, while this *C*–*V* method enables a more precise evaluation of the interface.

#### 3.2.2 Electrochemical Capacitance–Voltage (ECV)

**Figure 7:**
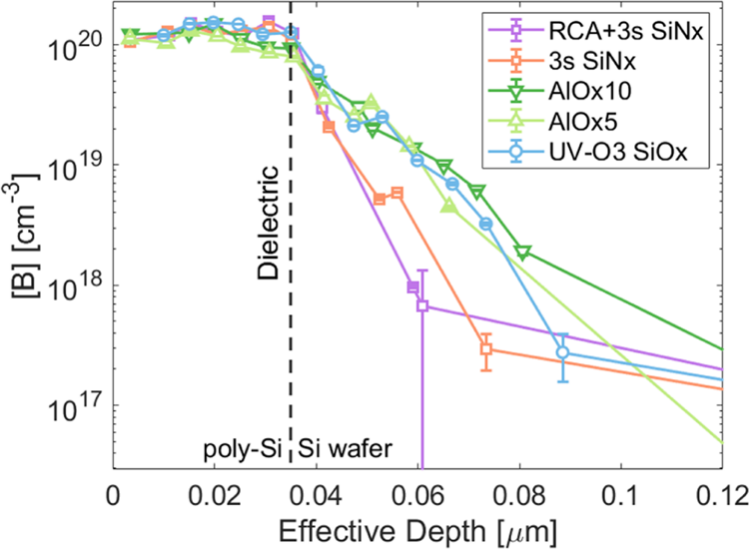
ECV doping profiles of poly-Si samples with SiO_*x*_, SiN_*x*_, and AlO_*x*_ nanolayers after 850 °C anneal.

The doping profiles of SiO_*x*_, SiN_*x*_, and AlO_*x*_ poly-Si contacts from Sample Set A were measured using ECV and are shown in Figure 7. SiO_*x*_ and AlO_*x*_ nanolayers have a diffusion depth between 90 and 120 nm, while the SiN_*x*_ has an apparent blocking effect, reducing the in-diffusion to 60–80 nm. The deep diffusion of the boron in SiO_*x*_ nanolayer samples can be linked to the high diffusivity/solubility of boron in SiO_x,_^60^ while phosphorus has a low diffusivity in the SiO_*x*_^61^ and therefore less in-diffusion is observed.^17^ The high boron concentration in the SiO_*x*_ results in increased strain and reduces the ability to passivate the dangling bonds at the Si surface.^62^ The SiN_*x*_ has a lower boron solubility and therefore could provide a better chemical passivation, possibly through utilizing a higher temperature or longer anneal. In addition, the steeper diffusion profile can increase the field-effect passivation across the dielectric and limit the Auger recombination in the device.^63^ We observe here that the SiN_*x*_ nanolayer brings an additional blocking effect to B-diffusion compared to SiO_*x*_ and AlO_*x*_. Thus, a SiN_*x*_ nanolayer could facilitate the optimization of p^+^ poly-Si contacts toward high surface passivation, high field-effect passivation, and low Auger recombination in the B-diffused region.

### 3.3 Improved Passivation

**Figure 8:**
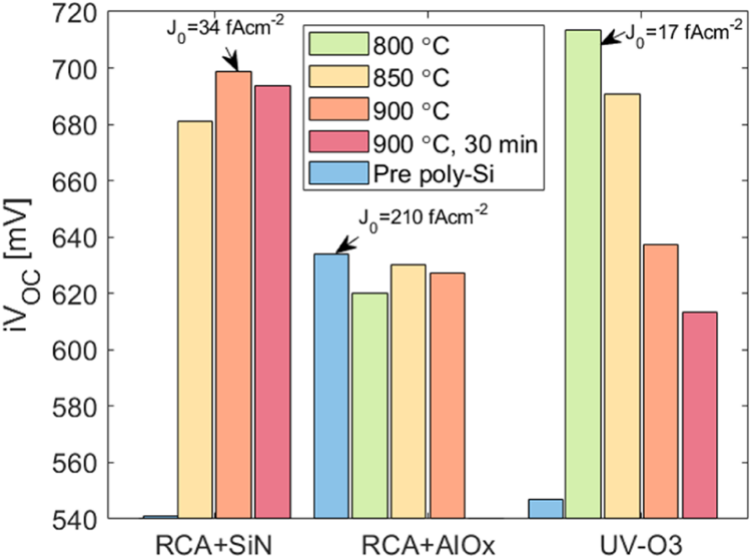
*iV*_OC_ for symmetrical RCA2 + SiN_*x*_ and RCA2 + AlO_*x*_ poly-Si structures with a range of annealing temperatures. The samples are measured after hydrogenation. The “pre poly-Si” measurement indicates the highest lifetime obtained without the poly-Si after a hot plate anneal. The hot plate anneal temperature was 600 °C for the RCA2 + SiN_*x*_ and UV–O_3_ samples and 350 °C for the RCA2 + AlO_*x*_ sample. The single-sided dark saturation current density, *J*_0_, is indicated for the best passivation achieved for each dielectric.

The *C*–*V* and ECV results elucidated possible paths to improve the passivation quality of the AlO_*x*_ and SiN_*x*_ contacts. The high charge obtained for the RCA2 + AlO_*x*_ sample make this an attractive option and a lower anneal temperature may be preferable to prevent the AlO_*x*_ breaking up, reducing its pinhole density. The RCA2 + SiN_*x*_ layer showed some promise in Sample Set A, with a significant enhancement possible if the uniformity was improved. In addition, the ability for SiN_*x*_ to block the boron can enable a higher thermal budget to be used. To improve the passivation, a new batch of samples was fabricated and termed Sample Set C. The effect of different poly-Si anneal temperatures was studied, focusing on the most promising nanolayers. In this iteration, handling of the wafers was minimized during RCA2 growth and subsequent depositions to improve passivation uniformity. Figure 8 shows the *iV*_OC_ for RCA2 + SiN_*x*_ and RCA2 + AlO_*x*_ layers at a range of annealing temperatures after hydrogenation. The *iV*_OC_ of samples with the dielectric but without a poly-Si layer is included here and referred to as “pre poly-Si”. These samples underwent a hot plate anneal in air before being measured.

**Figure 9:**
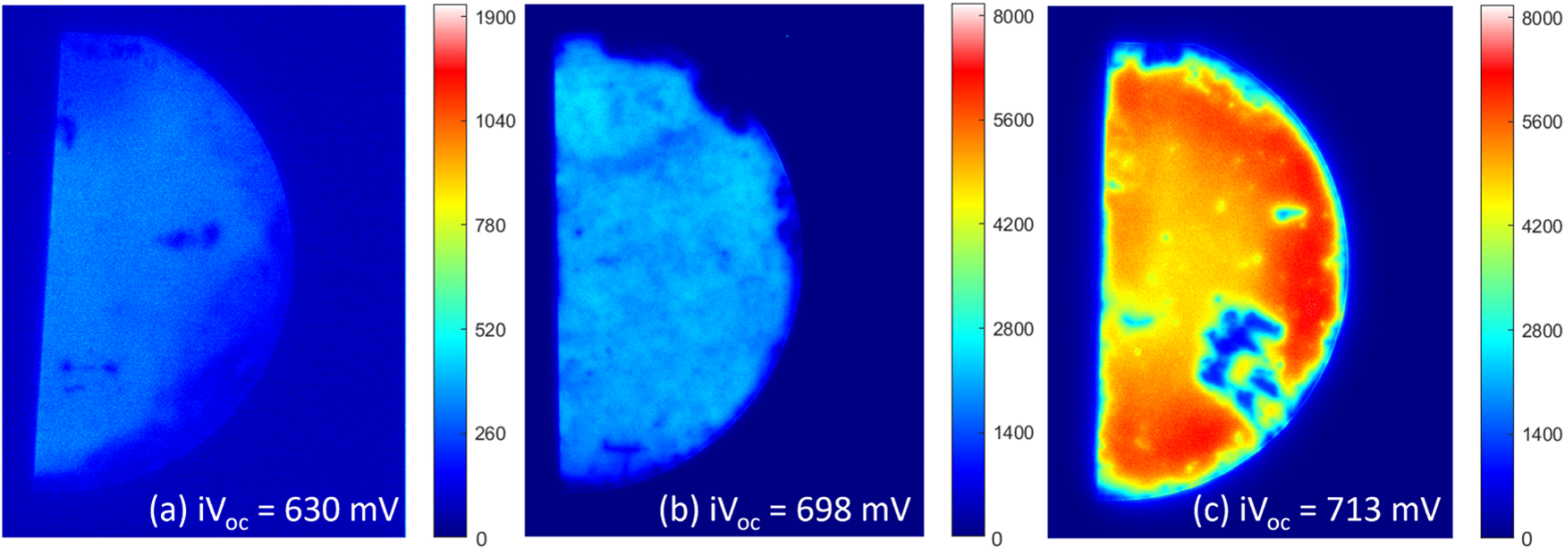
PL images for (a) RCA2 + AlO_*x*_ layers after 850 °C anneal, (b) RCA2 + SiN_*x*_ after 900 °C anneal, and (c) UV–O_3_ after 800 °C.

The RCA2 + SiN_*x*_ sample shows an increase in the passivation quality, with a maximum *iV*_OC_ of 698 mV after the 900 °C anneal. It also shows excellent thermal stability, with high *iV*_OC_ maintained across different anneal conditions. The UV–O_3_ passivation, on the other hand, drops significantly after 800 °C, indicating it is less stable to process variability. Figure 9 shows the corresponding PL image for the optimum anneal condition of each dielectric. A substantial improvement in the homogeneity of the passivation has been achieved for the SiN_*x*_, compared to Figure 5b. This shows that PECVD deposition is a viable method for achieving highly uniform passivation of nanolayer dielectrics across a full wafer. An improvement is also seen in the AlO_*x*_ nanolayers compared to Sample Set A, where lifetime was not measurable. However, only moderate *iV*_OC_ is achieved at present with AlO_*x*_ poly-Si contacts. Interestingly, the AlO_*x*_ nanolayers perform better than SiN_*x*_ and SiO_*x*_ before poly-Si deposition, but this does not translate to the final contact structure. This indicates that poly-Si deposition and high-temperature annealing may damage the Si/AlO_*x*_ interface. Recent work by Grant et al.^32^ shows that at temperatures up to 600 °C the high charge density remains in the nanolayer, however, at temperatures above 500 °C the chemical passivation begins to decrease. This may become more severe at the >800 °C temperatures in poly-Si contacts. We also observed some blistering in the poly-Si of the AlO_*x*_ samples, covering <0.5% of the total area (Figure S8). This may contribute to the poor lifetime and increase scatter in the results.

The resistivity of the samples from Sample Set C was measured and is included in the Supporting Information (Figure S2). The contact resistivity was notably higher for the SiN_*x*_ and AlO_*x*_ samples, while ρ_c_ for the UV–O_3_ samples was consistent compared to the previous results. It is expected this is due to slight variation in the dielectric thickness of the ALD and PECVD layers between batches. Further explanation is included in the Supporting Information. Optimization of the processing flow is required to achieve the best passivation at an acceptably low resistivity. This may include a more streamlined process flow to minimize the time between dielectric deposition and a-Si deposition. In the case of the thin SiN_*x*_ layers, it may be possible to combine its deposition with the a-Si in a single PECVD chamber. Such prospects will be explored in future work.

### 3.4 Efficiency Potential of Contacts Integrated into Full Device Structures

**Figure 10:**
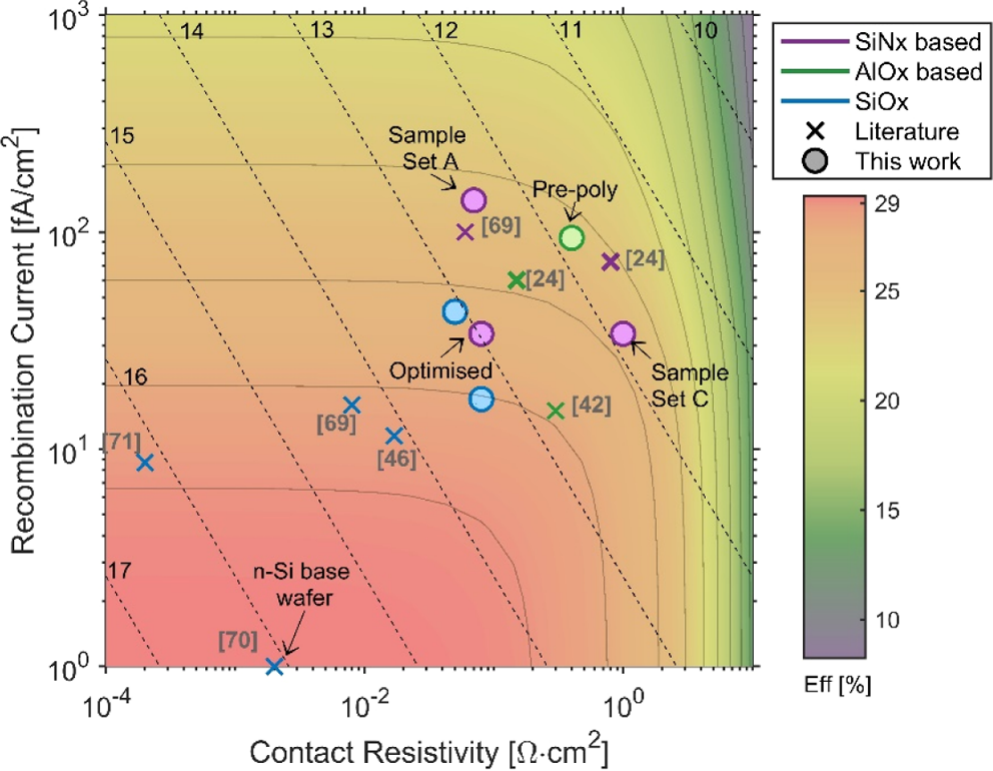
Contact selectivity of p^+^ poly-Si passivating contacts in the work compared to literature values.^22,37,43,49,68,69^ In instances where only *iV*_oc_ was reported this was converted to *J*_0_ using ref (44). The selectivity is shown as black dashed lines, while the gray lines indicate efficiency contour lines. The maximum potential efficiency is simulated using PC1D for a 110 μm, 1 Ω·cm p-type wafer with perfect light trapping and electron contact.

SiN_*x*_ and AlO_*x*_ passivation layers have been implemented into p^+^ poly-Si contacts. The RCA2+SiN_*x*_ stacks showed the most promise in the poly-Si contact structure with *iV*_OC_ approaching 700 mV and a single sided *J*_0_ of 34 fA/cm^2^. This is among the best passivation achieved for a SiN_*x*_, SiO_*x*_N_*y*_, or SiO_*x*_ + SiN_*x*_ stacks in p^+^ poly-Si contacts, bettering the *iV*_OC_ of 690 mV achieved by Reichel et al.^22^ Feldmann et al. achieved a similar *iV*_OC_ of 695 mV for a nitrided plasma oxide.^17^ As with SiO_*x*_ contacts, n^+^ poly-Si contacts using SiN_*x*_ nanolayers have achieved higher levels of passivation.^22,64,65^Figure 10 compares the best results from this work to literature values for p^+^ poly-Si contacts using SiN_*x*_, AlO_*x*_ or SiO_*x*_ nanolayers. The contact selectivity indicated by the black dashed lines is a metric devised by Brendel et al.^66^ as a figure of merit for passivating contacts. It is noted that the best p^+^ poly-Si contacts were fabricated on n-type Si wafers, with an enhanced FEP. Studies using SiO_*x*_ far outnumber those using any other passivation layers. Thus, it is unsurprising that the best results are still observed from extensive optimization of the processing for SiO_*x*_.^49,67,68^

The AlO_*x*_ structures in this work have so far been less effective as poly-Si contacts despite showing the highest levels of passivation prior to poly-Si deposition. The cause of this passivation loss is suspected to be due to blistering seen after the poly-Si anneal (Figure S8). Blistering is caused by hydrogen outgassing during the high-temperature anneal, which can damage the Si/dielectric interface.^70^ Initial tests show some improvement in the passivation when the AlO_*x*_ layer undergoes a low-temperature anneal before the poly-Si processing (Figure S8). Optimization of this step could prevent blistering and enable the full potential of the AlO_*x*_ nanolayers to be exploited.

Understanding the nanolayer dielectrics has enabled us to adapt our processes to maximize the capabilities of each dielectric (within only two experimental iterations) and understand where they differ from the ubiquitous SiO_*x*_. The capped *C*–*V* method introduced in this paper provides a tool to obtain *Q*_f_ and *D*_it_ to high accuracy and precision in highly conductive nanolayer dielectrics. The PECVD SiO_*x*_ capping layer provides an insulating layer to enable good-quality *C*–*V* measurements, while the Si/dielectric interface remains the primary influence of the measured capacitance signal. The additional processing of the SiO_*x*_ layer could influence the Si/dielectric interface, due to the high concentration of hydrogen in the PECVD SiO_*x*_ altering the chemical passivation of the interface, thus leading to an underestimation of the *D*_it_. This is not necessarily a problem, as any full contact structure will undergo a hydrogenation step. In fact, with optimization of the SiO_*x*_ anneal, the hydrogenation of the interface could be maximized to give an idea of the minimum *D*_it_. The results from the capped *C*–*V* show good agreement with the estimated values of *D*_it_ and *Q*_f_ from SPV measurements in previous work.^23^ It is noted that the technique cannot be applied to the dielectrics in the full poly-Si structure, however, the information it provides is still valuable.

Significant improvements in the passivation properties were made after only two process iterations (Sample Set A and C), while further optimization is required to combine this with low contact resistivity. The rapid progress can be partly attributed to the understanding obtained from the capped *C*–*V* process in combination with the T–J–V and PL images. These results highlight the importance of developing a complete understanding of the nanolayer dielectric in the poly-Si contact structure. Despite having a thickness of <10 atomic layers, these interlayers govern the contact’s resistivity and passivation quality. A deeper understanding of these layers is extremely beneficial to balancing the passivation and transport requirements of the contact. It is vital in the search for ever-higher power conversion efficiencies in solar cells.

## 4 Conclusions

The development of highly efficient hole contacts is required to complement the already established electron-selective TOPCon structure and further increase the efficiency of poly-Si based Si solar cells. In this work, we investigated SiN_*x*_ and AlO_*x*_ based stacks as the passivating nanolayer in p^+^ poly-Si contacts. The SiN_*x*_ and AlO_*x*_ offered potential advantages over SiO_*x*_ including a lower barrier height for hole tunneling (SiN_*x*_ and AlO_*x*_), an advantageous boron diffusion profile (SiN_*x*_), or an enhanced FEP due to negative charge at the Si surface (AlO_*x*_). We fabricated poly-Si contacts with low contact resistivity for AlO_*x*_ and SiN_*x*_ layers and, using T–J–V, we provide the first demonstration of the conduction mechanisms in hole-selective poly-Si contacts using alternative dielectrics. This proved it is possible to fabricate a purely tunneling SiN_*x*_ p^+^ poly-Si contact. However, thus far their passivation still does not match the UV–O_3_ SiO_*x*_ reference. We found the AlO_*x*_ samples to have the best passivation prior to poly-Si deposition, partly due to a high charge density of 3 × 10^12^ q/cm^–2^. However, the surface passivation did not improve after processing of the p^+^ poly-Si layer on top of AlO_*x*_, in contrast to SiN_*x*_ and SiO_*x*_. Further analysis is required to determine if the observed blistering is the cause of the limited passivation obtained with AlO_*x*_, and to develop a pathway to increase the passivation in a complete contact structure. Recombination and charge transport measurements helped determine that a high pinhole density and nonuniformity hampered the passivation quality of the RCA2 + SiN_*x*_ samples, not a high positive charge. With improved processing, we obtained a significant improvement in the passivation quality, obtaining a highly uniform PECVD SiN_*x*_ nanolayer with a single-sided *J*_0_ of 34 fA/cm^2^. Using direct RF PECVD, we showed the potential of growing tunneling nanolayers, demonstrating a particularly appealing prospect for streamlined processing of hole-selective polysilicon contacts.

## Data Availability

All data created during this research and published in this article is openly available from the Oxford University Research Archive and can be downloaded free of charge from http://ora.ox.ac.uk.

## Supporting Information Available

Additional resistivity measurements including detailed fitting for an example temperature-dependent current–voltage measurement and measurements for Sample Set C; photoluminescence and minority carrier lifetime measurements for Sample Set A and C, including the fitting for the extraction of *J*_0_; XPS measurements used for stoichiometry analysis of the AlO_*x*_ and SiN_*x*_ films; micrograph images of blistering observed in the AlO_*x*_ samples from Sample Set C and the improvement in *iV*_oc_ observed when the AlO_*x*_ underwent an additional annealing step before poly-Si deposition (PDF)
