# Supplementary material for: Hole-Selective SiNx and AlOx Tunnel Nanolayers for Improved Polysilicon Passivating Contacts
Source: ACS Appl Energy Mater. 2024 Nov 14;7(22):10259–70. doi: 10.1021/acsaem.4c01170 (PMC11600395; doi:10.1021/acsaem.4c01170)
Supplement: Supplementary file 1 — ae4c01170_si_001.pdf [file ae4c01170_si_001.pdf]

Hole-selective SiN_x and AlO_x Tunnel Nanolayers for Improved Polysilicon Passivating Contacts: Supporting Information

Shona McNab[a,c], Audrey Morisset[b], Sofia Libraro[b], Ezgi Genç[b], Xinya Niu[a], Jack E. N. Swallow[a], Peter Wilshaw[a], Robert S. Weatherup[a], Matthew Wright[a], Franz-Josef Haug[b], Ruy S. Bonilla*[,a]

[a] Department of Materials, University of Oxford, Oxford, OX1 3PH, United Kingdom
[b] School of Engineering, Ecole Polytechnique Fédérale de Lausanne, Neuchâtel, CH-2000 Switzerland
[c] School of Photovoltaic and Renewable Energy Engineering, University of New South Wales, 2052, Australia

*Corresponding author e-mail: sebastian.bonilla@materials.ox.ac.uk

1 Resistivity Measurements

Detailed fitting of temperature dependent J-V measurements.

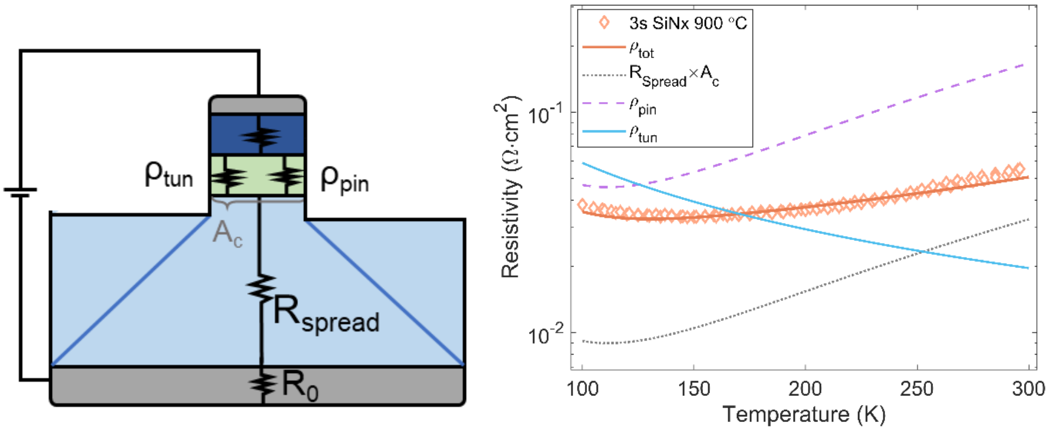

Figure S1: a) Sample schematic with an equivalent circuit diagram for the T-IV measurement fitting. b) 3s SiNx + poly-Si structure annealed at 900°C from sample set B. The tunnelling, pinhole, and wafer spreading resistance have been separated to show the comparative contributions to the total resistivity.

Resistivity measurements from Sample Set C are shown in Figure S2, using an identical structure as in Figure S1a. It is noted that the samples processed for the resistivity measurements did not undergo the SiN_x deposition and firing step. The SiN_x and AlO_x layers show higher resistivity compared to the previous batch. Tunnelling contacts are highly susceptible to thickness variations, thus it is expected that a slight increase in the dielectric thickness compared to Sample Set A has caused this increase. The temperature-dependent JV measurements are also included in Figure S2 and confirm a slight increase in dielectric thickness, combined with a significant reduction in the pinhole density compared to Sample Set A. The slight increase in the dielectric thickness may inhibit the pinhole formation. Thus, the lower tunnelling current, is combined with a lack of pinholes to prevent a low resistivity contact from forming.

The cause of the thickness increase could be due to inconsistencies in the ALD or PECVD deposition. However, this is not expected to be the case as the samples are processed individually, while the high resistivity is seen throughout. It is instead suspected that the thickness increase occurs during storage between the dielectric and poly-Si deposition steps. The dielectric layers are known to be sensitive to moisture and humidity prior to poly-Si deposition [1], so the storage conditions and length of time between dielectric and poly-Si deposition are likely important. Unfortunately processing restraints required the samples to be shipped between dielectric and poly-Si deposition. The samples were transported in a desiccating/vacuum atmosphere to try and reduce film growth/changes but this may have been insufficient. Ideally poly-Si deposition should immediately proceed dielectric deposition, or the samples should be packaged in an N2/desiccating/vacuum atmosphere.

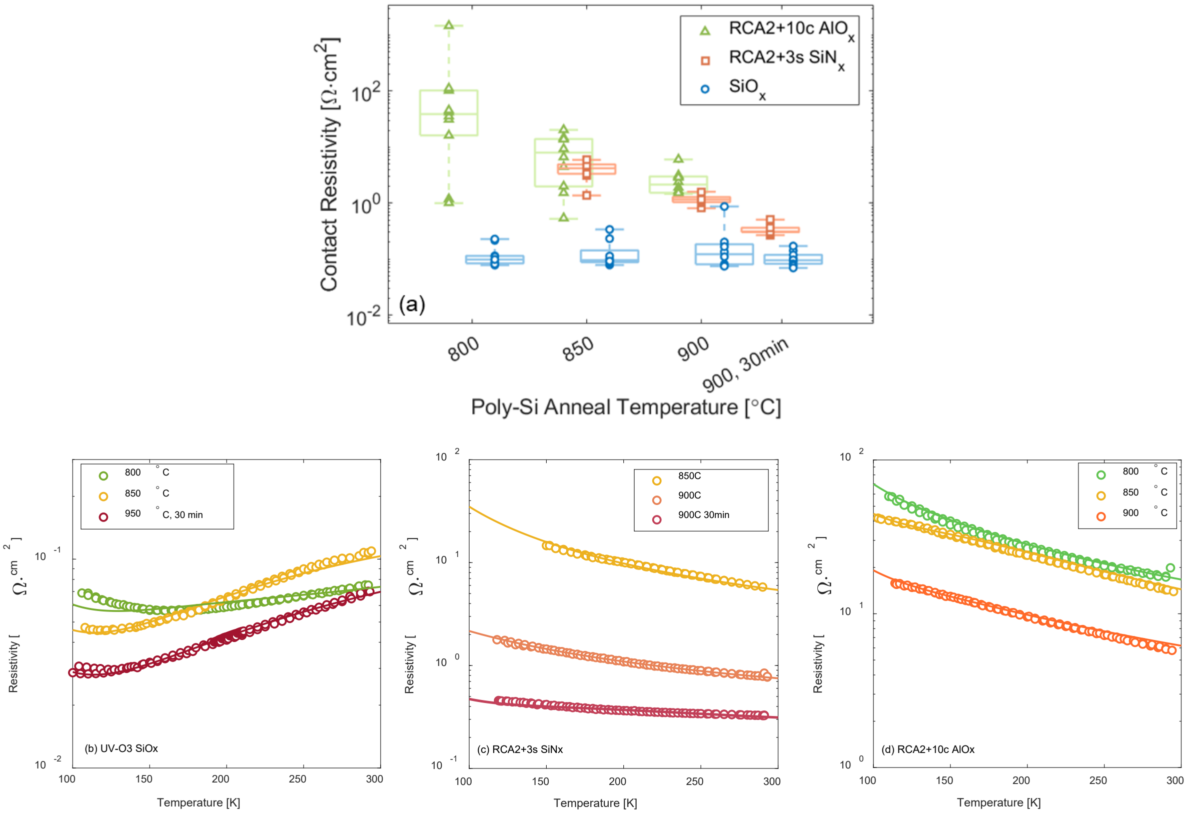

Figure S2: a) Contact resistivity of Sample Set C for RCA2+SiN_x, RCA2+ALO_x and UV-O_3 poly-Si contacts for a range of anneal conditions. Temperature-dependent resistivity measurements for b) UV-O3 SiOx, c) RCA2+3s SiNx and d) RCA2+10c AlOx

Table S1: Fitting parameters for T-JV measurements of Sample Set C.

	Dielectric	T [°C]	VBO [eV]	t_{diel} [nm]	Φ_b [eV]	N_{pin} [cm^{-2}]
**Set C**	UV-O_3 SiO_x	800	4.3	1.17	0	3.5×10^7
	UV-O_3 SiO_x	850	4.3	1.25	0	7×10^7
	UV-O_3 SiO_x	900, 30min	4.3	1.2	0	1.3×10^8
	RCA2 + 3 s SiN_x	850	1.4	2.34	0.01	0
	RCA2 + 3 s SiN_x	900	1.4	2.15	0	0
	RCA2 + 3 s SiN_x	900, 30min	1.4	2.05	0	3×10^6
	RCA2 + 10c $AlOx_x$	800	3.5	1.75	0.01	1.5×10^4

RCA2 + 10c $AlOx_x$	850	3.5	1.71	0.02	5×10^4
RCA2 + 10c $AlOx_x$	900	3.5	1.67	0.01	8×10^4

2 PL and Lifetime measurements

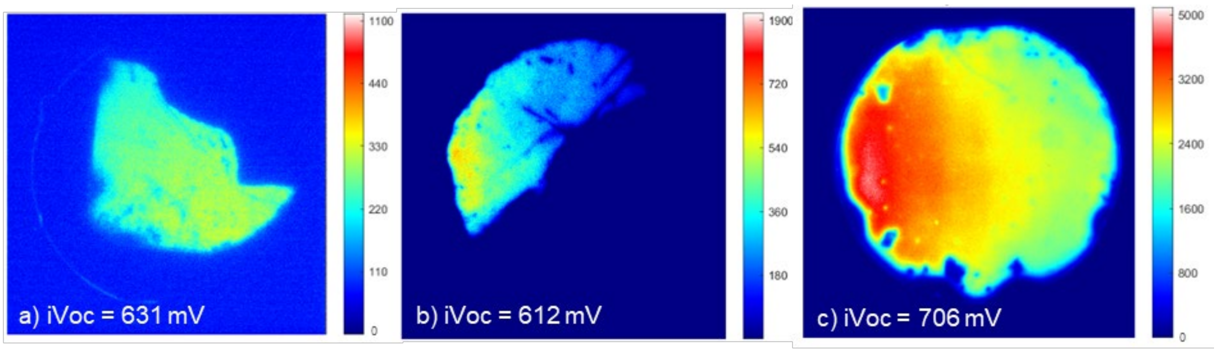

Figure S3: Photoluminescence images of additional samples in Sample Set A, measured after hydrogenation. a) SiN_x, b) RCA+SiN_x c) UV-O_3 SiO_x.

The iVoc was extracted from the effective lifetime measured from photoconductance decay. Figure S4 shows the minority carrier lifetime corresponding to the iV_{oc} data in Figures 4 and 8. In addition, the surface recombination current, J_0 was calculated for the condition which showed the highest levels of passivation for each dielectric. The J_0 was calculated using [2] and the resulting plots are shown in Figure S5.

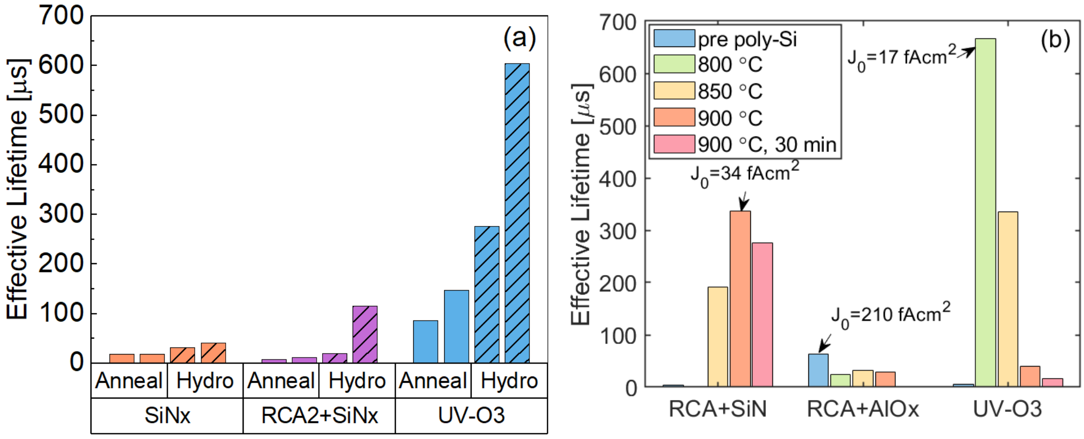

Figure S4: Minority carrier lifetime measurements at an excess carrier density of 10^{15} cm^{-3} corresponding to the iVoc at 1 Sun for a) Sample Set A shown in Figure 4, and b) Sample Set B in Figure 8.

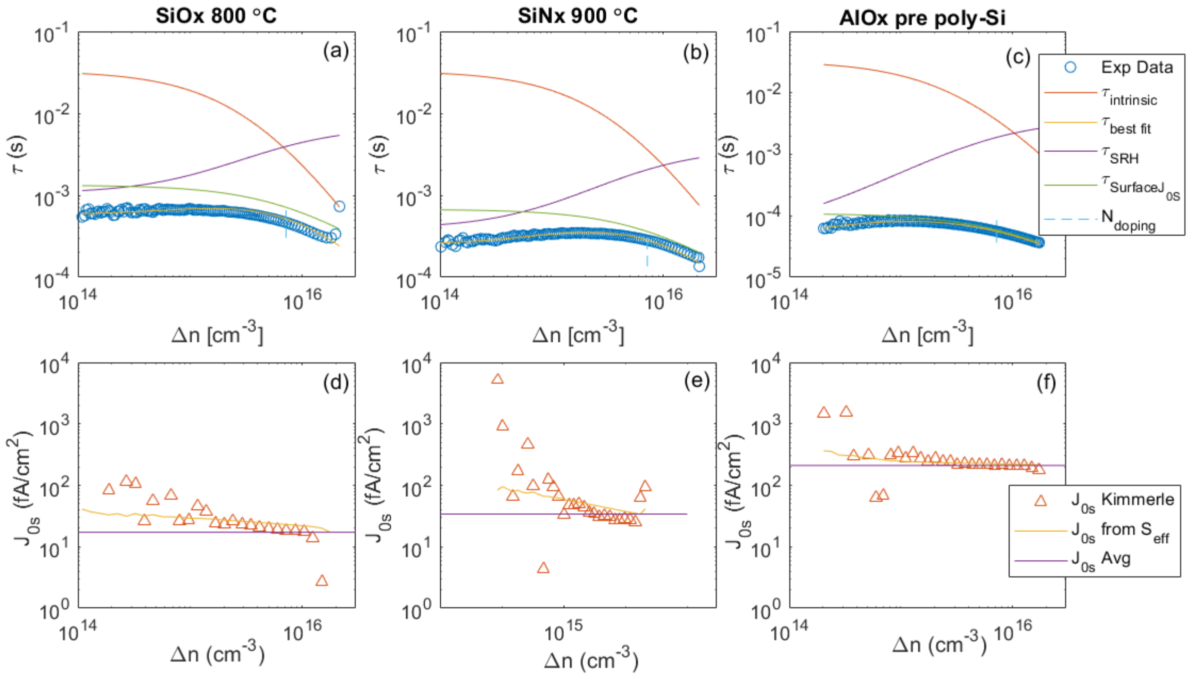

Figure S5: a)-c) Fitting of effective lifetime curves into contributions from surface and bulk effects. d)-f) Fitting of J_0 using [2].

3 XPS Stoichiometry Analysis

XPS measurements were performed using two XPS systems, a Thermo Scientific K-Alpha (TSK) and a Phi Versaprobe III (PhiV). The TSK uses a monochromatic Al Kα X-ray (1.487 keV) source with a 400 µm beam diameter, a beam angle of 54°, and an electron take-off angle of 90°. A pass energy of 20 eV is used for the core level spectra, while a higher pass energy of 50 eV is used for the valance band onset due to the small signal at this binding energy. The PhiV uses a monochromatic Al Kα X-ray (1.487 keV) source with a 100 µm beam diameter, a beam angle of 90°, and an electron take-off angle of 45°. A pass energy of 26 eV is used for all scans. Bulk samples underwent sputtering to remove any undesired surface layers. Sputtering is carried out over a 3×3 mm area using Ar^+ ion-milling at a potential of 1 kV. Peaks fitting is performed in CasaXPS using Shirley backgrounds and pseudo-Voigt (LA50) line shapes. Al2p and Si2p have closely spaced spin-orbit components, which was considered for fitting the Si^0 where this could be well resolved, but was not considered for the other components. The signal obtained from the core levels is converted into atomic% using the relative sensitivity factors (RSF). These were taken from the CasaXPS reference library [3] for the TSK and Moulder's X-ray photoelectron handbook [4] for the PhiV. Separate reference tables were required due to the different geometries of the two instruments, but a good agreement between the instruments is seen for the bulk films. Figure S6 shows an example peak fitting for an a) AlO_x and b) SiN_x nanolayer. The peak areas are indicated, after correcting for the relative sensitivity.

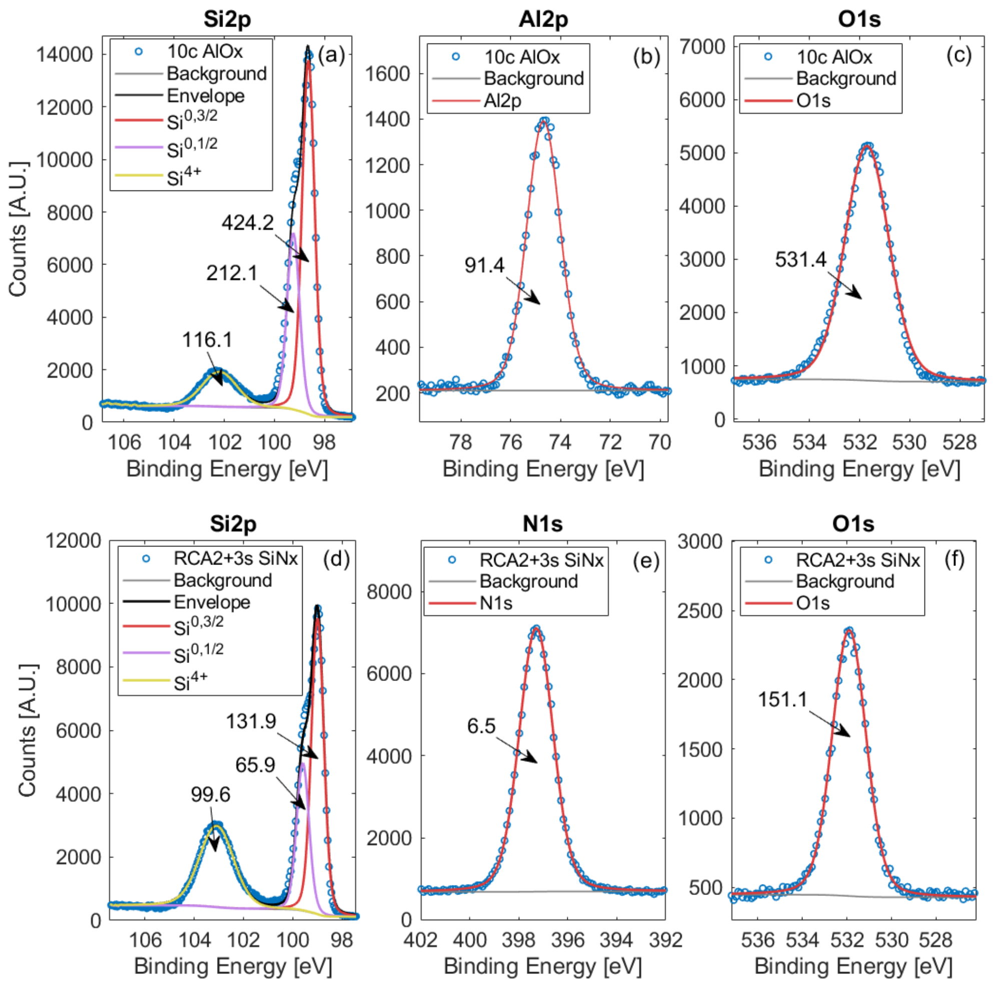

Figure S6: Example peak fitting of XPS data a)-c) for a 10 c AlO_x nanolayer and d)-f) for a RCA2+3 s SiN_x nanolayer on Si. Values indicate the RSF corrected Area (Raw Area/RSF).

Figure S7 compares the composition of AlO_x and SiN_x films. Only the bonded silicon (Si^{4+}) is considered in the stoichiometry analysis, with the metallic silicon (Si^0) assumed to be entirely from the silicon substrate. The bulk AlO_x is close to stoichiometric, while the nanolayers show increasingly high oxygen content as the thickness is reduced. The trend is reversed when the RCA2 oxide interlayer is included. Despite there being an intentional oxide, the total Al:O ratio increases. This is due to ALD deposition being highly sensitive to the chemical nature of the substrate surface. The H-terminated Si surface inhibits Al deposition, while the O-terminated Si can promote Al deposition. As the number of cycles is short in these nanolayers this has a significant influence on the films. The PECVD SiN_x composition is shown in Figure S7b. The signal from the silicon wafer (doublet at ~99 eV) is removed for the analysis of the SiN_x composition. The bulk layers are silicon rich, which can provide a better passivation of the surface [5]. A considerable amount of oxygen is also present in the bulk films. This is reduced after sputtering, but it is not fully removed. The oxygen concentration increases as the film thickness is reduced and for the samples which were annealed in air. The Si:N ratio is constant for a 10 s deposition but the 5 s SiN_x after anneal and the sample with RCA2+3s SiN_x show a substantial drop in the nitrogen concentration.

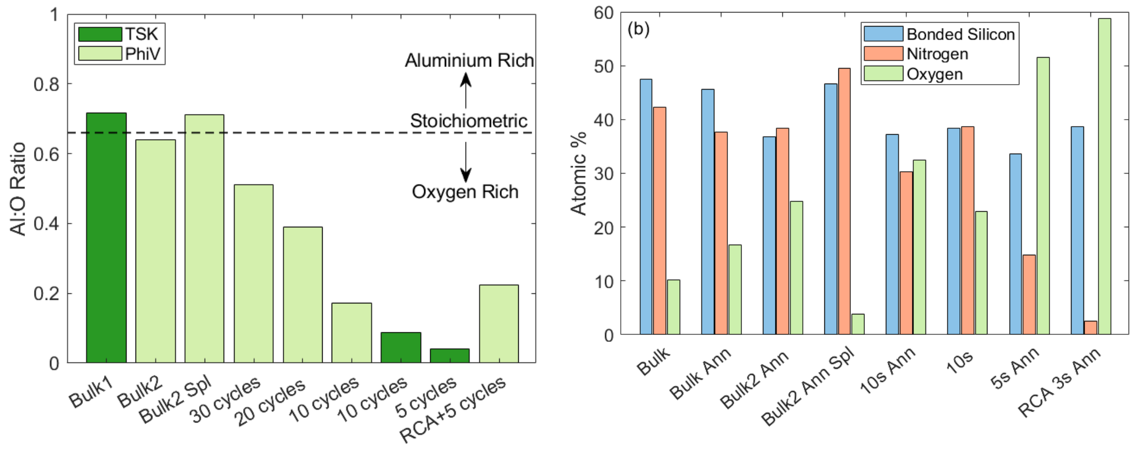

Figure S7: XPS stoichiometry of a) AlO_x nanolayers and b) SiN_x nanolayers on Si.

4 Blistering of AlO_x poly-Si contacts

Blistering was observed in the AlO_x contacts after poly-Si deposition and SiNx hydrogenation. The blistering may occur due to outgassing of hydrogen during the anneal, to mitigate this, an AlO_x sample was run with a 10 min, 350 °C anneal performed before the poly-Si deposition. This saw some improvement in lifetime, however, blistering was still observed. A longer or higher temperature anneal may lead to further improvements in the passivation of the AlO_x nanolayers.

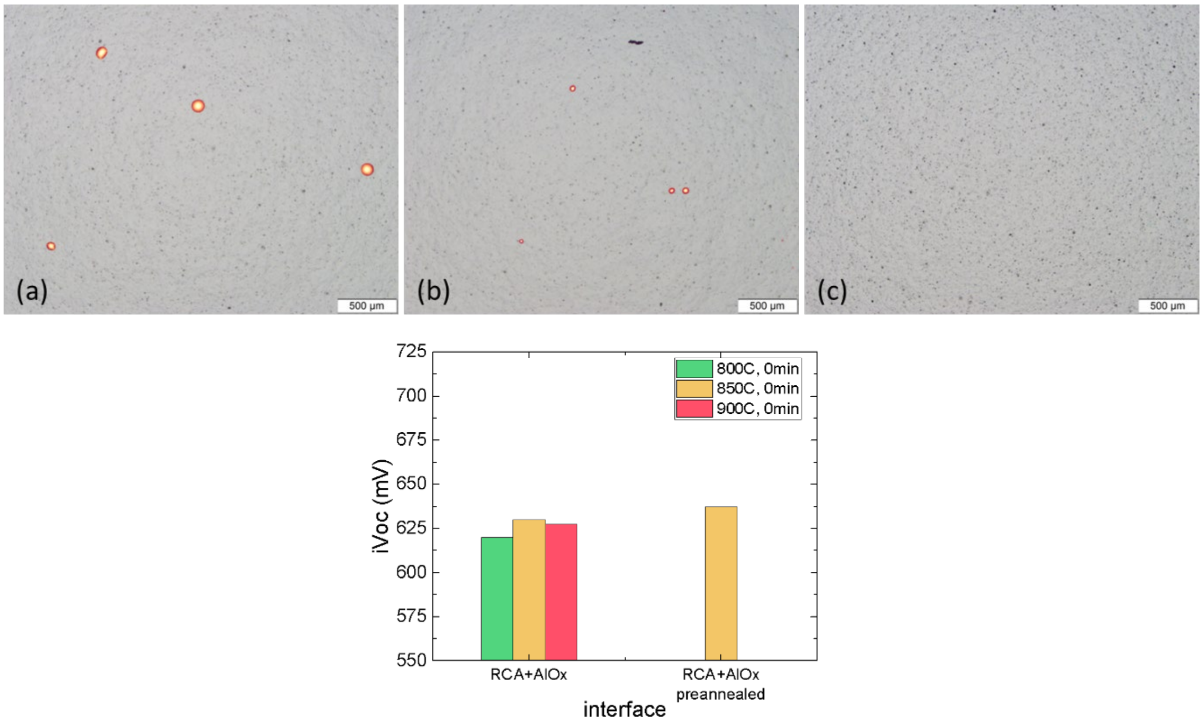

Figure S8: Blistering of RCA2+AlO_x layers a) without pre-anneal (area fraction <0.5%), b) with a pre-anneal (area fraction <0.05%), c) Micrograph of RCA+SiN_x contact showing no blistering d) iV_{OC} measurements of RCA+10c AlO_x poly-Si contacts without and with a pre-anneal.

References

(1) McNab, S.; Niu, X.; Khorani, E.; Wratten, A.; Morisset, A.; Grant, N. E.; Murphy, J. D.; Altermatt, P. P.; Wright, M.; Wilshaw, P. R.; Bonilla, R. S. SiNx and AlOx Nanolayers in Hole Selective Passivating Contacts for High Efficiency Silicon Solar Cells. *IEEE J Photovolt* **2022**, 1–11. https://doi.org/10.1109/JPHOTOV.2022.3226706.

(2) *Oxford Interfaces Lab: SintonAnalysis*. https://github.com/OxfordInterfacesLab/SintonAnalysis (accessed 2022-08-18).

(3) Fairley, N. *CasaXPS Manual*; 2009.

(4) Moulder, J. F. *Handbook of X-Ray Photoelectron Spectroscopy: A Reference Book of Standard Spectra for Identification and Interpretation of XPS Data*; Chastain, J., Ed.; Physical Electronics Division, Perkin-Elmer Corporation, 1992.

(5) Lelièvre, J. F.; Fourmond, E.; Kaminski, A.; Palais, O.; Ballutaud, D.; Lemiti, M. Study of the Composition of Hydrogenated Silicon Nitride SiNx:H for Efficient Surface and Bulk Passivation of Silicon. *Solar Energy Materials and Solar Cells* **2009**, *93* (8), 1281–1289. https://doi.org/10.1016/J.SOLMAT.2009.01.023 .
